# Variation across a wheat genetic diversity panel for saccharification of hydrothermally pretreated straw

**DOI:** 10.1186/s13068-017-0914-x

**Published:** 2017-10-02

**Authors:** Samuel R. A. Collins, David R. Wilson, Graham K. Moates, Andrea L. Harper, Ian Bancroft, Keith W. Waldron

**Affiliations:** 1 The Biorefinery Centre, Quadram Institute Bioscience, Norwich Research Park, Colney, Norwich, NR4 7UA UK; 2 Department of Biology, University of York, Wentworth Way, Heslington, York, YO10 5DD UK

**Keywords:** High-throughput screening, Bioethanol, Lignocellulose, Saccharification, Fermentation, Wheat straw, Pretreatment

## Abstract

**Background:**

Wheat straw forms an important, reliable source of lignocellulosic biomass for use in second-generation ethanol production. However, there is limited understanding of the variation in quality of straw from current breeding cultivars, and studies on such variation have generally employed suboptimal pretreatments. There is also a degree of confusion regarding phenotypic characteristics relevant to optimising the enzymatic saccharification of cellulose after suitable pretreatments for biorefining compared with those which determine good ruminant digestibility. The aim of this study has been to (a) evaluate and compare the levels of glucose enzymatically released from straw obtained from 89 cultivars of winter wheat after optimised hydrothermal pretreatments and (b) identify the underlying phenotypic characteristics relevant to enhanced glucose production with special reference to the ratios of constituent tissue types.

**Results:**

Optimised pretreatment involved hydrothermal extraction at 210 °C for 10 min. Using excess cellulases, quantitative saccharification was achieved within 24 h. The amount of glucose released ranged from 192 to 275 mg/g. The extent of glucose release was correlated with (a) the level of internode tissue (*R* = 0.498; *p* = 6.84 × 10^−7^), (b) stem height (*R* = 0.491; *p* = 1.03 × 10^−6^), and (c) chemical characteristics particular to stem tissues including higher levels of cellulose (*R* = 0.552; *p* = 2.06 × 10^−8^) and higher levels of lignin *R* = 0.494; *p* = 8.67 × 10^−7^.

**Conclusions:**

In order to achieve maximum yields of cellulosic glucose for second-generation ethanol production, a predisposition for wheat to produce cellulose-enriched internode stem tissue, particularly of longer length, would be beneficial. This contrasts with the ideotype for ruminant nutrition, in which an increased proportion of leaf tissue is preferable.

**Electronic supplementary material:**

The online version of this article (doi:10.1186/s13068-017-0914-x) contains supplementary material, which is available to authorized users.

## Background

Lignocellulose makes up approximately half of the world’s biomass [1]. A significant tonnage comprises underutilised field waste produced in agriculture. For example, the annual production of wheat straw in the UK is approximately 6 M tonnes, and in Europe it is approximately 74 M tonnes [2]. Hence, there is considerable interest in exploiting such residues as sources of cellulosic glucose for the production of bioethanol and/or chemicals. The economic exploitation of such field wastes would potentially add value to crop production and would help avoid the development of a potential food–fuel dilemma by exploiting the inedible components.

Wheat breeding has predominantly focused on optimising the yield and quality of the wheat grain by reducing the amount of biomass associated with the non-grain components, and increasing the level of harvestable material by reducing the incidence of, e.g. lodging [3], susceptibility to phytopathogens [4] and increasing grain size [5]. More recently, there has been increasing interest in using similar approaches to improve the enzymatic digestibility of wheat straw, for example, as animal feed or for conversion to bioethanol. Jensen et al. [6] used an in vitro enzymatic digestion process to assess the varietal variation in wheat straw digestion. They found that shorter cultivars were more digestible, and suggested that this was due to the larger proportion of less lignified leaf tissues. Other studies have also demonstrated that wheat leaf tissues are more readily susceptible to enzymatic saccharification than stem tissues after a range of pretreatments including alkali or hot acid [7], sodium carbonate [8], and HTP microplate hydrothermal treatments [9, 10]. Such results lead to the conclusion that a higher proportion of leaf tissue would result in a higher yield of ethanol. However, Lindedam et al. [11] reported a positive correlation between wheat height and straw conversion, but found no association with ratios of anatomical parts, and concluded that the quality of the various component parts was more important than the ratios between them. The studies on cultivar variation generally used relatively low (suboptimal) severity pretreatments, presumably due to limitations in the high-throughput technology available, or in an unspecified attempt to identify cultivars which produce straw that requires lower pretreatment severities. However, glucose released would then be a function of both recalcitrance of the lignocellulosic material as well as the total amount of cellulosic glucose present, thereby confusing the interpretation of data.

Recently, Collins et al. [12] used multivariate modelling of FT-IR spectroscopy to assess wheat straw tissues and compositions from 90 cultivars of UK winter wheat. The study demonstrated that a dominant source of compositional variation within straw of modern wheat varieties was due to the variation in ratios of leaf and internode tissues, and that higher levels of cellulosic glucose were associated with a higher internode-to-leaf ratio. Thus, whilst leaves might be more readily digestible after lower severity pretreatments, more cellulose is available from stems.

In order to provide further clarity and to further interpret the range of contrasting hypotheses presented in the scientific literature, the aim of this study has been to (a) evaluate and compare variation in the enzymatic saccharification of straw from 89 cultivars of winter wheat after optimised hydrothermal pretreatments and (b) identify the underlying phenotypic characteristics which promote saccharification yields with special reference to the ratio of the constituent tissues [12]. Such knowledge could lead to the improvement of cultivars for straw functionality. The prospect of creating valuable, renewable fuels and chemicals from the inedible straw could significantly increase the economic benefit of wheat production and reduce its overall carbon footprint.

## Methods

### Wheat straw samples

For development and optimisation of pretreatment conditions and enzymolysis, the work utilised winter wheat straw which was supplied by Norfolk Straw Ltd., Hill Farm, East Dereham, Norfolk NR20 3HB. The main study employed a panel of 89 cultivars of wheat grown and used previously [12]. The wheat cultivars were grown in the UK at KWS UK, Royston, Herts, and material was harvested in the summer of 2011. The field-grown wheat plants were cut at ground level and dried in perforated bags in air under ambient conditions. The grain was separated from the ‘waste stream’ tissues and the whole straw used for analysis. For each cultivar, three plants were harvested. These were left to dry for 2–3 months to ensure air dryness before further treatment.

### Sample homogenisation by milling

Wheat straw is a heterogeneous and highly structured material. Because the applied analysis methods use only small amounts of material, the straw was homogenised in order to facilitate representative sampling. The equilibrated air-dried (between 7 and 8% moisture) whole plants were milled with a J&K MF10 analytical sieve mill (IKA^®^-Werke GmbH & Co. KG; Janke & Kunkel-Str. 10; Staufen, Germany) to less than 250 μm. Any remaining material greater than 250 μm was re-milled for 7 min with a J&K A10 grinder with a water cooling jacket (IKA^®^-Werke GmbH & Co. KG; Janke & Kunkel-Str. 10; Staufen, Germany) to less than 250 μm (ensured by passing through a 250-μm sieve shaker). The milled powder was mixed thoroughly before being sampled for pretreatment and saccharification studies.

### Particle size analysis

Particle size was determined using an LS13320-MW Laser Diffraction Particle Size Analyser (multi-wavelength version) with universal liquid module (ULM) [Beckman Coulter, Inc., Brea CA, 92821-6232, USA].

### Moisture determination

Moisture content of the milled material was determined by drying on a METTLER PM200 IR balance with METTLER LP16 drying unit (METTLER-TOLEDO, company HQ: Greifensee, Switzerland).

### Hydrothermal pretreatment of milled wheat straw

The milled wheat straw was further cryogenically milled to a fine powder in a 6970EFM Freezer mill (SPEX Sample Prep, Stanmore, UK) using the following optimised procedure:

(a) Precool by immersing the loaded cuvettes in liquid nitrogen for 1 min; (b) precool by placing the loaded cuvettes into the freeze-mill grinding chamber (cooled to liquid nitrogen temperatures but not in direct contact with the liquid nitrogen) for 20 min; (c) perform milling operation with a running time of 3 min at a rate of 8 cycles per second; (d) re-cool for 4 min; (e) repeat steps (c) and (d) for 4 more cycles; and (f) allow samples to equilibrate to room temperature overnight before opening cuvettes.

For the milling of wheat cultivars, the freeze-mill (FM) cuvettes were loaded with 3.6 g of material mixed from each of 3 replicate plant samples (1.2 g each) obtained from a wheat diversity panel reported previously [12].

Hydrothermal pretreatment was performed using a BIOTAGE^®^ Initiator + reactor (Biotage AB, Box 8, 751 03, Uppsala, Sweden). Duplicate freeze-milled straw samples (each 0.75 g air-dry weight [12]) were weighed into 20-ml microwave pressure tubes. To each of these was added 14.25 ml Milli-Q^®^ water to give a 5% (w/w) suspension. Whilst continually stirring at 900 rpm, the samples were thermally treated to the required severity factors (allowing 55–70 s for initial heating period). At the end of the treatment, the samples were cooled with compressed air to ambient and then centrifuged at 1811×*g* for 10 min to yield a clear supernatant. Samples of soluble supernatant were taken and stored at −20 °C until required. The remaining solid, insoluble residue was quantitatively transferred to a 50-ml Falcon tube by washing with Milli-Q water. The volume was brought to 40 ml, and the sample re-centrifuged (2465×*g*, 20 min, at 4 °C). After removing the supernatant (which was retained), the pellets were re-washed and centrifuged 4 times after which the final pellet was stored at −20 °C until required.

### Saccharification of pretreated wheat straw: 15-ml scale

These studies were carried out to evaluate the effect of pretreatment severity on saccharification and production of fermentation inhibitors, and enzyme digestion time courses. Pretreated wheat straw (winter wheat; washed residue from 750 mg original dry matter) was re-suspended in 0.1 M sodium acetate (pH 5.0) to a total volume of 14 ml in a 15-ml Falcon tube. To this was added Cellic CTec2 (50 FPU/g original straw substrate air-DWt) such that the final substrate loading was 5% (w/v). The tubes were mixed and then placed in a Gerhardt Thermoshake incubator at 50 °C/120 rpm for 5 days. At the end of the incubation period, the tubes were cooled on ice, centrifuged at 2830*g* for 15 min, and then the supernatants recovered and frozen prior to further analysis for levels of glucose and xylose monosaccharides.

### Separate saccharification of pretreated wheat straw: 1-ml scale

These studies were carried out using microtubes on 96-position plates to evaluate the effect of enzyme concentration and saccharification of cultivars. Calculations were carried out on the basis of the original air-dried sample weight.

Pretreated and washed wheat straw pellets derived from 750 mg freeze-milled wheat straw samples were re-suspended into 30 ml Milli-Q water in 50-ml Falcon tubes. They were then maintained as a uniform suspension by stirring with a magnetic stirrer. Using wide-aperture 1.0-ml pipette tips, 0.84 ml replicate samples of suspended particles were quantitatively pipetted into 1.0-ml screw-top Matrix tubes (TrakMates 2D barcoded storage, Thermo Scientific Matrix; Fisher Scientific UK Ltd, Bishop Meadow Road, Loughborough, LE11 5RG). After centrifugation, an aliquot of 90 µl supernatant was removed from each sample matrix tube using a multichannel pipette in order to make space in the tube to allow the addition of an aliquot (90 µl) of buffer solution containing concentrated amounts of enzyme and thiomersal for saccharification. The concentrated amounts of buffer, enzyme, and thiomersal were chosen such that the final concentrations of each were correct for the 0.84 ml of slurry/buffer mix finally remaining in the tube. This addition, by multichannel pipette, initiated saccharification. To each Matrix tube were added two autoclaved glass balls. The Matrix tubes were capped, inverted, and vortex mixed after which they were incubated in a 25 °C temperature-controlled room on an orbital shaker plate (insert details) set at 120 rpm, fixed in position with each plate on its side to allow lateral movement of the substrate (and glass balls) along each tube from end to end.

### Quantification of Fermentation inhibitors

Pretreatment-derived supernatants were re-centrifuged at 2465*g* and 200 μl of the supernatant was filtered using a syringe filter (0.2 µm, Whatman International Ltd, Maidstone, UK), and injected into vials. The concentrations of the fermentation inhibitors 2-furfuraldehyde (2-FA), 5-hydroxymethylfurfural (5-HMF), and the organic acids (formic and acetic acid) were analysed by an HPLC using a Flexar LC instrument (PerkinElmer, Seer Green, Bucks., UK) equipped with refractive index and photo diode array detectors (outputting chromatograms at 210, 280, and 325 nm wavelengths) in series. The analyses were carried out using an Aminex HPX-87H organic acid analysis column (Bio-Rad Laboratories Ltd, Hemel Hempstead, UK) operating at 65 °C with 0.004 mol/l H_2_SO_4_ (Sigma-Aldrich) as the mobile phase at a flow rate of 0.6 ml/min.

### Quantification of reducing sugars and ethanol

#### HPLC

Samples were centrifuged, filtered, measured using an HPLC fitted with an Aminex HPX-87H organic acid analysis column with an RI detector [13]. Xylose, glucose, and ethanol were detected and quantified against external standards.

#### GOPOD

Glucose concentrations were quantified using a glucose-specific kit (GOPOD, Megazyme, Bray, Republic of Ireland) using a scaled approach developed previously for sugar analysis [14]. Substrate and enzyme controls were included wherever necessary.

### Analysis of cell wall composition

Cell wall composition data were taken from Collins et al. [12].

## Results

### Sample milling

The controlled milling of the wheat straw was essential. The operation of the BIOTAGE small-scale microwave pretreatment apparatus required that the 20 ml volumes were stirred to maintain uniform suspensions during heating in order to prevent hot spots and associated tube failure particularly at the higher pressures. In addition, uniform suspensions of pretreated slurries were required to accurately dispense pretreated substrate into 1-ml Matrix tubes using multichannel pipettes and liquid handling robotics. Preliminary experiments with freeze milling apparatus demonstrated that the rate and the extent of biomass disruption were considerably influenced by the initial particle size, the quantity of the input material, the time of pre-freezing in liquid nitrogen, and the duration and hammer frequency of the mill. For example, the effect of milling duration of < 3 mm wheat straw on particle size distribution is shown in Additional file 1: Figure S1a, b, and the effect of milling < 1 mm wheat straw in Additional file 1: Figure S1c, d. The optimised conditions chosen for freeze milling involved pre-milling to a particle size range of less than 250 µm using a mesh mill [12] followed by pre-freezing 3.6 g samples and then subjecting them to 5 × 3 min freeze milling cycles with an impact rate of 8 cycles per second. The fine powder produced had a mean particle size of 45 µm (SE = 1 µm; particle size profile is shown in Additional file 1: Figure S1e, f).

Particle disruption is a form of “pretreatment” in its own right and will influence the extent of enzymatic saccharification of lignocellulose by increasing the accessibility of enzymes to cellulose and hemicelluloses, increasing the surface area-to-volume ratio of the material, and helping to stir and mix the sample, thus enhancing fluid movement. Particle sizes considered in previous studies range from mm through to submicron. Vidal et al. [15] reviewed the influence of feedstock particle size down to 0.15 mm on lignocellulose conversion and indicated that particle size reduction could increase digestibility, but to a limit of about 50%, and thermochemical pretreatment was still required to optimise conversion. A similar effect was found by Wood et al. [16] who freeze-milled oilseed rape straw cultivars prior to assessing hydrothermal pretreatment and saccharification using a high-throughput approach based on Elliston et al. [17]. Extreme particle disruption has also been assessed. For example, Shutova et al. [18] demonstrated that the enzymatic hydrolysis of wood polysaccharides ground into ultrafine submicron particles (UFPs) increased the yield of sugars during enzymatic hydrolysis. Furthermore, Silva et al. [19] reported that ultrafine grinding using ball milling to 20 µm or less reduced cellulose crystallinity and increased glucose yield to the same order achieved by steam explosion due to an increase in surface area and then internal disruption. However, they provided no information on the steam explosion severity, and their data suggested that their yield of glucose released was in the order of 2/3 of that available indicating that both approaches were considerably suboptimal. Hence, whilst the freeze milling in the current study will have had an impact on saccharification, the results support the premise that this is limited, and that hydrothermal pretreatment is required to achieve quantitative saccharification. Thus, the milling of straw to particles with an average size of 45 µm provides a substrate which requires conventional hydrothermal pretreatment in order to be fully saccharified and is thus appropriate for screening approaches.

### Optimisation of pretreatment conditions

**Fig. 1:**
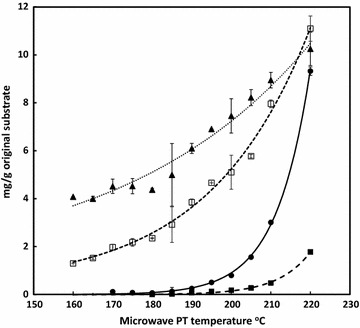
Effect of pretreatment severity on inhibitors released into liquor (mg/g original substrate). Symbols: Formic acid (filled triangle); 5-HMF (filled square); 2-FA (filled circle); Acetic acid (opened square) (*n* = 2)

**Fig. 2:**
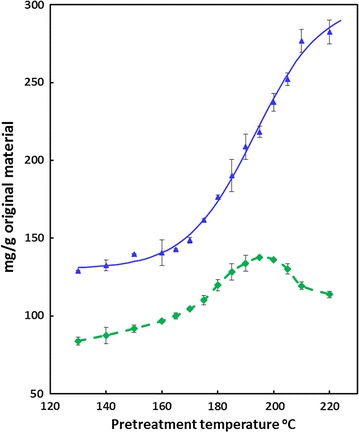
Effect of pretreatment severity on the enzymatic release of glucose (blue triangle) and xylose (green diamond) from wheat straw (*n* = 2)

Freeze-milled wheat straw was pretreated for 10 min at temperatures between 130 and 220 °C (duplicate samples) in a Biotage microwave reactor (see Methods). The pretreated material was washed thoroughly, re-suspended in water, and dispensed into 15-ml screw-capped reaction tubes for digestion studies. Breakdown products comprising furfurals and organic acids are shown in Fig. 1. The extent of digestibility of the pretreated material was evaluated by digesting with excess cellulase (Cellic CTec2; 50 FPU/g original substrate) at 50 °C for 5 days. The release of glucose and xylose is presented in Fig. 2 and shows that maximum glucose release required pretreatments at or above 210 °C for 10 min (Fig. 2). This is entirely consistent with studies on the impact of steam explosion severity on saccharification of wheat straw [20, 21]. The severe reduction in measurable xylose at above 190 °C will be due to thermal degradation as reported previously in hydrothermal pretreatment studies [22, 23]. This was accompanied by the rapid increase in the production of 2-FA (Fig. 1), a breakdown product from xylose, at the higher severities. Small quantities of 5-HMF were also produced. The levels of 2-FA and 5-HMF were consistent with earlier reports for pretreated wheat straw [24]. Similar trends have also been shown by Silva-Fernandes et al. [25].

Non-linear regression analysis (GENSTAT [26]) created an expected very simple sigmoidal curve for glucose release which accounted for 98.9% of the variance (Fig. 2).

### Optimisation of enzyme loading

**Fig. 3:**
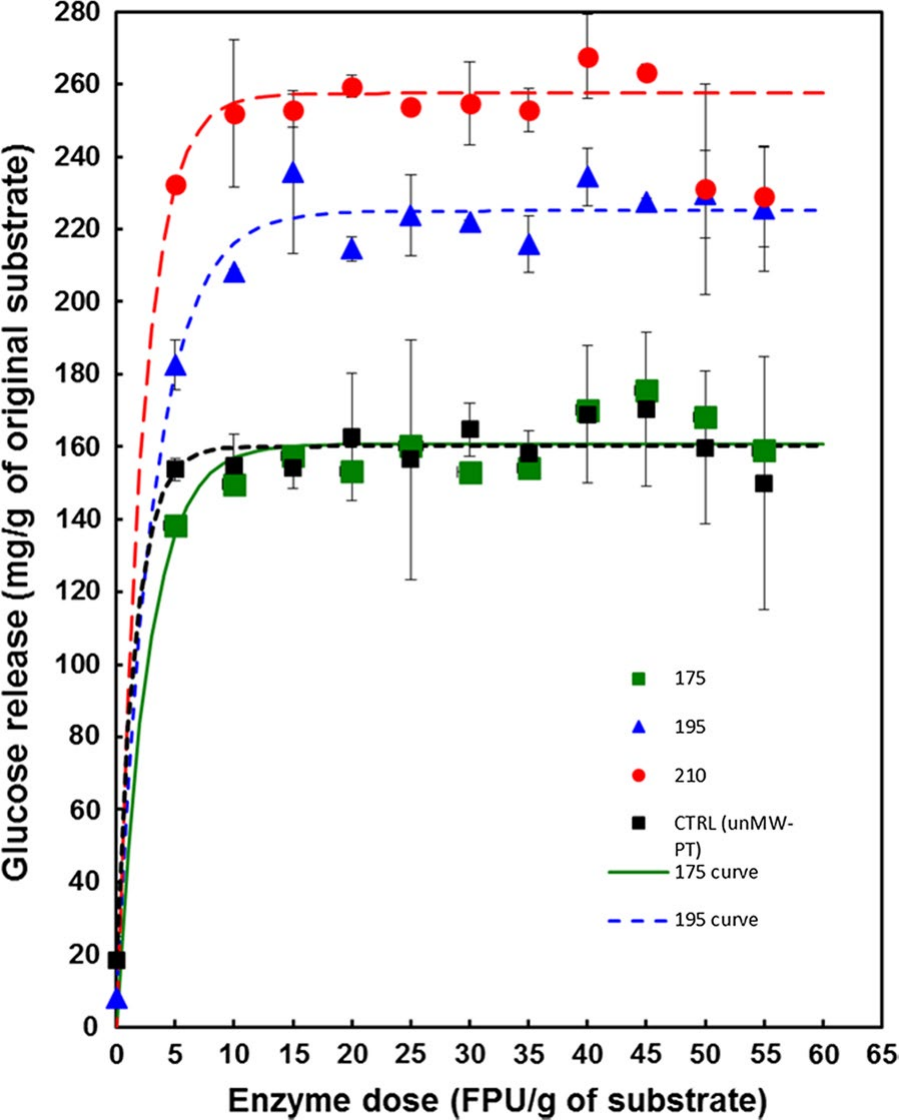
Effect of cellulase dose on the release of glucose from wheat straw pretreated at three different severities: Symbols: control (black squares); 175 °C for 10 min (green squares); 195 °C for 10 min (blue triangles); 210 °C for 10 min (red circles); *n* = 2

The amount of cellulase necessary for quantitative saccharification of pretreated wheat straw at a substrate concentration of 5% (w/v) was evaluated at three severities: 175, 195, and 210 °C (Fig. 3). In all cases, saccharification over a 96-h period had reached a plateau at and above the enzyme loadings of 10–15 FPU/g original straw dry matter. This is consistent with the enzymolysis of hydrothermally pretreated wheat straw reported by Holopainen-Mantila et al. [27] although they used a 1% (w/v) substrate loading and a different enzyme source. Saccharification of straw pretreated at 175 °C resulted in yields similar to those produced from freeze-milled, non-pretreated straw (Fig. 3).

### Optimisation of saccharification time

**Fig. 4:**
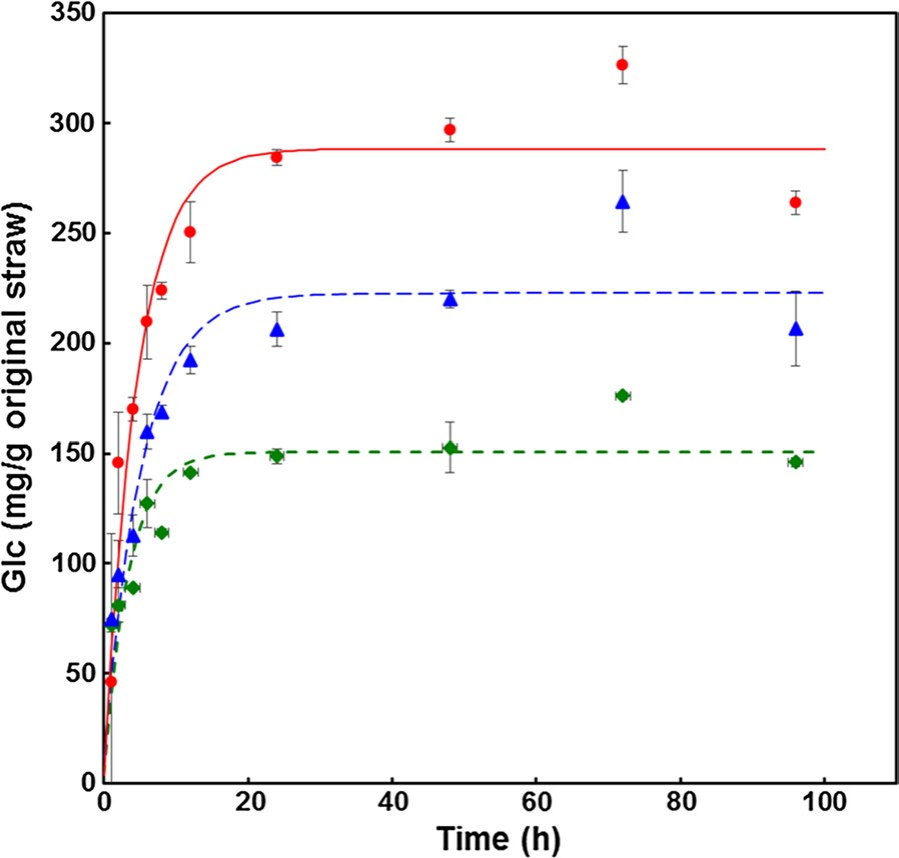
Time course of glucose release from wheat straw pretreated at 175 °C (green diamonds), 195 °C (blue triangles), and 210 °C (red circles) for 10 min, saccharified with excess cellulase (30 FPU/g original material); *n* = 2

In order to ensure that sufficient and reliable digestion was achieved, wheat straw pretreated at 175, 195, and 210 °C was saccharified with excess cellulase (30 FPU/g original material). At all severities, the digestion had reached completion after 24 h (Fig. 4).

### Saccharification of straw from across a genetic diversity panel

On the basis of the above results, the pretreatment and saccharification conditions chosen for screening the 89 cultivars of wheat involved freeze-milling the material, pretreatment at 210 °C for 10 min, saccharification at 50 °C for 96 h with an enzyme loading of 30 FPU/g, and a substrate loading of 5% (w/v) in the presence of Thiomersal. For each pretreated cultivar, a single saccharification was performed. Glucose released was measured in duplicate using the GOPOD method. In addition, the pretreatment liquors were evaluated for levels of key fermentation inhibitors 2-FA and 5-HMF.

The profiles of variations in the inhibitors produced and glucose released across the cultivars are shown in Additional file 2: Figures S2a, b, Additional file 3: Figure S3. The amount of glucose released showed a wide spread from 192 mg/g straw to 275 mg/g. Similar ranges in glucose release were shown by Bekiaris et al. [28] from 203 wheat varieties over many locations and by Bellucci et al. [29] who studied saccharification of 100 cultivars of wheat.

**Fig. 5:**
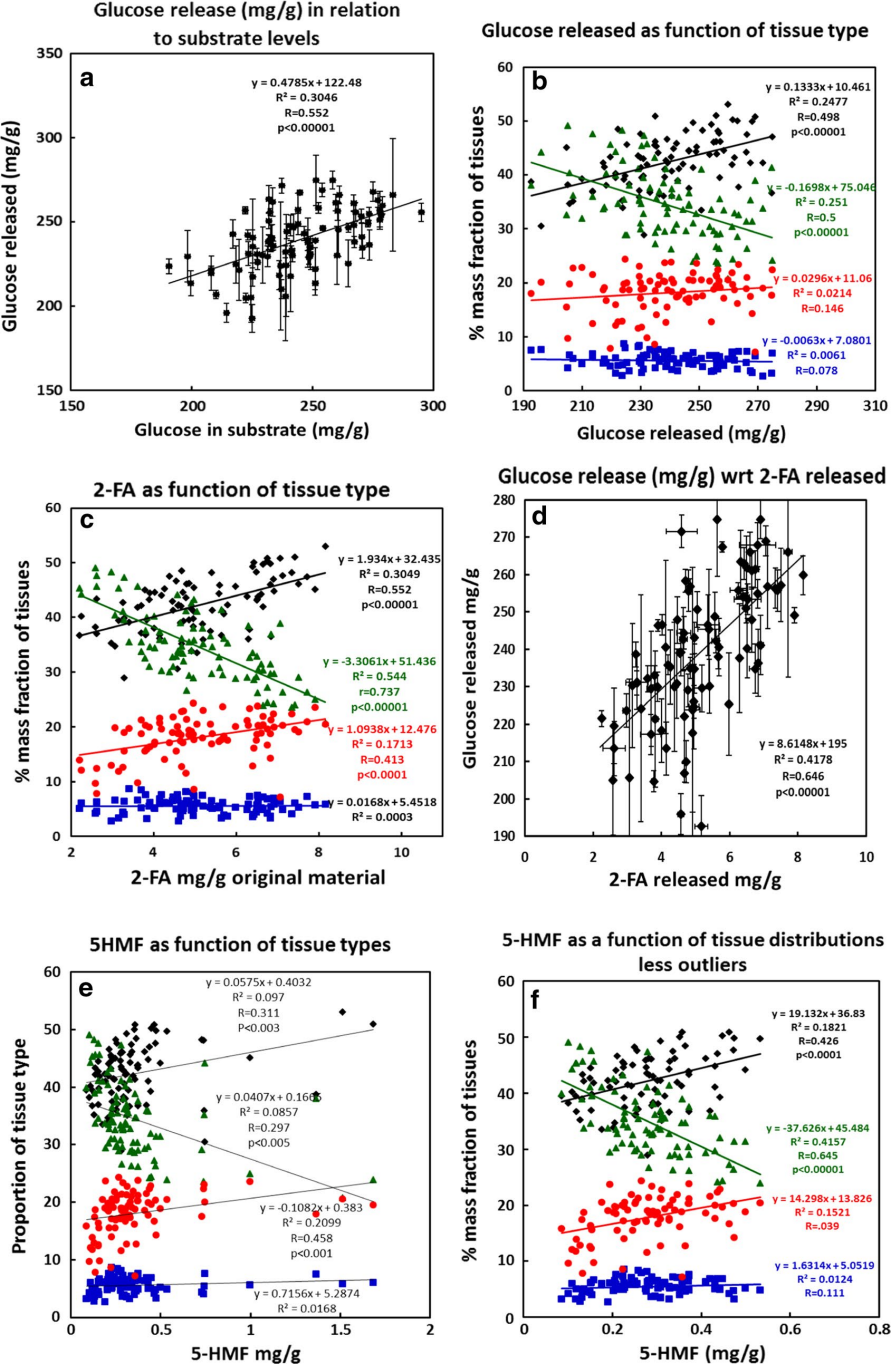
Analysis of wheat straw from 89 cultivars of UK (predominantly winter) wheat. **a** Correlation of glucose released by saccharification of optimally pretreated straw with glucose present in the original straw (*n* = 3); **b** glucose released from saccharified straw as a function of the % mass fractions of component tissues (*n* = 3); **c** 2-FA released during pretreatment of straw as a function of the mass fractions of component tissues (*n* = 3); **d** 5-HMF released during pretreatment of straw (not including “outliers” as a function of the mass fractions of component tissues (*n* = 3); **e** as for **d** but including “outliers”; **f** straw height (less ear) as a function of the mass fractions of component tissues (*n* = 3). Standard deviations (SD) are not shown in **b**–**f** to enhance clarity. For glucose release, SD are shown in **a**. Key to tissues: stem (black diamonds), leaf (green triangles), ear (red circles), and node (blue squares). Note that in **a** and **b** data for cultivar “Humber” were not available and in **c**–**e** data for cultivar “Istabraq” were not available

**Fig. 6:**
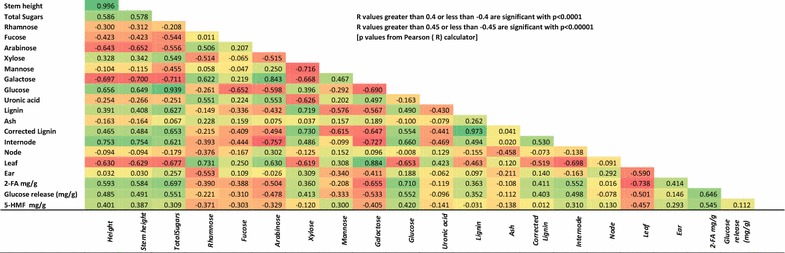
Correlation table

Previously, we demonstrated that the levels of cell wall sugars in wheat straw biomass were highly dependent on the ratios of the different tissue types (internode, node, leaf, and ear) [12]. Therefore, the current saccharification yields and amounts of fermentation inhibitors produced were compared with the previously published compositional and phenotype data. Graphical representation of selected associations is shown in Figs. 5 and a correlation table of these data sets is shown in Fig. 6. The enzymatic release of glucose was found to be strongly correlated with the levels of glucose present in the substrate (*R* = 0.552; *p* = 2.06 × 10^−8^ Figs. 5a, 6). In keeping with this, the release of glucose was also found to be strongly dependent on the ratios of the component tissue types (Fig. 5b; Fig. 6). Specifically, the release of glucose was positively correlated with the level of internode tissue (*R* = 0.498; *p* = 6.84 × 10^−7^) and stem height (*R* = 0.491; *p* = 1.03 × 10^−6^) and negatively correlated with the level of leaf tissue (*R* = −0.501; *p* = 5.72 × 10^−7^). The glucose release was correspondingly negatively correlated with the levels of cell wall galactose (*R* = −0.533; *p* = 7.56 × 10^−8^) and arabinose (*R* = −0.478; *p* = 2.17 × 10^−6^; Fig. 6), reflecting their higher levels in the leaf tissues [12], and positively correlated with the levels of lignin, reflecting the higher levels of lignin in the internode stem tissues (Fig. 6, *R* = 0.494; *p* = 8.67 × 10^−7^). There was no significant correlation of glucose release with the levels of either node or ear tissues.

The pretreatment release of fermentation inhibitors was also found to be related to the compositional and phenotypic variation. 2-FA production was positively correlated with internode stem tissue (Fig. 5c; *R* = 0.552; *p* = 2.06 × 10^−8^) and negatively with leaf tissue (Fig. 5c; *R* = −0.737; *p* = 1.78 × 10^−8^), and this corresponds to the different levels of xylose in these tissues that would have been degraded during pretreatment [12]. Interestingly, the release of glucose was also highly correlated with the release of 2-FA (Fig. 5d; *R* = 0.646; *p* = 8.13 × 10^−12^) probably reflecting the relationship with pretreatment-induced degradation of xylan from the cellulose. This is supported by the observation of Bekiaris et al. [28] where enzymatic glucose release was also correlated positively with xylose release. 5-HMF production was also subjected to similar trends (Fig. 5e), but these were only statistically significant in relation to leaf tissues (Fig. 6). However, these trends were very much clearer after the removal of some outlying variants which produced higher levels of 5-HMF than the rest of the population (compare Fig. 5e, f).

## Discussion

The present study shows clearly the dominating influence of the stem-to-leaf tissue ratio in determining the level of enzymatic glucose release after optimised pretreatment. This reflects the impact of tissue ratios on the levels of cellulosic glucose present in the biomass described previously by Collins et al. [12]. The previously reported correlation between stem internode tissue and levels of lignin [12] is consistent with the positive correlation between glucose release and levels of lignin (Fig. 6) in this study. This observation is seemingly contradictory to established wisdom that lignin is inhibitory to saccharification of lignocellulose [30]. However, in this study, the adverse impact of lignin has effectively been annulled by the optimised pretreatment such that the lignin no longer presents a physicochemical barrier to hydrolysis. Saccharified glucose is essentially dependent on the amount of cellulose to be hydrolysed.

The impact of tissue ratios contrasts with the results of Lindedam et al. [11]. In their study, there was no significant cultivar correlation between leaf-to-stem ratios and sugar yields and, additionally, lignin was negatively correlated with saccharification yields. These key differences may be readily explained by the differences in pretreatment severity. In the Lindedam et al.’s [11] study, the much lower severity pretreatment was considerably suboptimal and thus allowed the lignin component to have a large influence on saccharification to the extent that it overrode any other influences such as tissue ratios.

In keeping with the current study, Lindedam et al. [11] observed that plant height had a positive impact on cellulosic glucose release. Since they did not observe any significant relationship between stem-to-leaf ratio and either saccharification or height, they suggested that this could reflect differences in plant structure which might be more open in expanded tissues. This is a reasonable hypothesis that requires further investigation. However, their study involved only 20 different cultivars and it is possible that such relationships were difficult to detect. In this study of 89 cultivars, height was highly correlated with the proportion of stem tissues and negatively with leaf (*p* < 0.00001 for both, Fig. 6). Interestingly, higher stem-to-leaf ratios were not only associated with longer stems but also the strength of the second internode (see [3] and Fig. 6 in [12]). This is probably due to a higher level of maturation and secondary cell wall development of the second internode tissues in longer stems.

There has been considerable debate in the literature regarding the importance of the stem-to-leaf ratio in relation to ruminant digestibility [6] and saccharification [11]. Researchers have often observed that the leaf tissues are more digestible; hence, a higher leaf-to-stem ratio would be seemingly more beneficial. This is relevant for studies on animal nutrition. However, in order to maximise the yields of enzymatically released glucose, then so long as the pretreatment is sufficient (as in this study), the stem tissue should be maximised since it has higher levels of cellulosic glucose than leaf tissue [12]. This conclusion is consistent with the observations of Lindedam et al. [11]. Consequently, the ongoing preference for cultivation of semi-dwarf wheat varieties is limiting the saccharification potential of modern straw residues.

## Conclusions

Total straw from 89 cultivars of UK winter wheat was milled, hydrothermally pretreated at 210 °C for 10 min, and then enzymatically saccharified. The amount of glucose released ranged from 192 mg/g to 275 mg/g, demonstrating that field residues are not uniform in their saccharification potential. Increased glucose release was clearly correlated with (a) the level of internode tissue (*R* = 0.498; *p* = 6.84 × 10^−7^), (b) stem height (*R* = 0.491; *p* = 1.03 × 10^−6^), and (c) chemical characteristics particular of stem tissues including higher levels of cellulose (*R* = 0.552; *p* = 2.06 × 10^−8^) and higher levels of lignin (*R* = 0.494; *p* = 8.67 × 10^−7^). In order to achieve maximum yields of cellulosic glucose for second-generation ethanol production, a predisposition for wheat to produce cellulose-enriched internode stem tissue, particularly of longer length, would be beneficial. This contrasts with the ideotype for ruminant nutrition, in which an increased proportion of leaf tissue is preferable.

## Additional files

**Additional file 1: Figure S1.** Particle size distributions of milled wheat straw. (a) [log scale for particle size] and (b) [linear scale for particle size]: 3 mm straw freeze milled for 1 (green circle), 2 (blue square) and 3 (red triangle) minutes at 8 cycles per second. (c) [log scale for particle size] and (d) [linear scale for particle size]: < 1 mm straw (blue circle) and after freeze milling for 1 min at 8 cycles per second (green triangle). (a) [log scale for particle size] and (b) [linear scale for particle size]: 3.6 g samples of < 0.25 mm straw before and after being pre-frozen and then subject to 5 × 3 min freeze milling cycles with an impact rate of 8 cycles per second.

**Additional file 2: Figure S2.** Production of fermentation inhibitors during pretreatment of 89 cultivars of wheat (a) 2-FA and (b) 5-HMF; means and standard deviations; n = 2.

**Additional file 3: Figure S3.** yields of saccharified glucose from pretreated straw of 89 different wheat cultivars; means and standard deviations; n = 2.

## Abbreviations

HTP: high throughput
FT-NIR: Fourier transform near infrared
FM: freeze-mill
FPU: filter paper units
2-FA: 2-furfuraldehyde
5-HMF: 5-hydroxymethylfurfural
CTRL: control
