# Supplementary figures and images for: Variation across a wheat genetic diversity panel for saccharification of hydrothermally pretreated straw
Source: Biotechnol Biofuels. 2017 Oct 2;10:227. doi: 10.1186/s13068-017-0914-x (PMC5625621; doi:10.1186/s13068-017-0914-x)

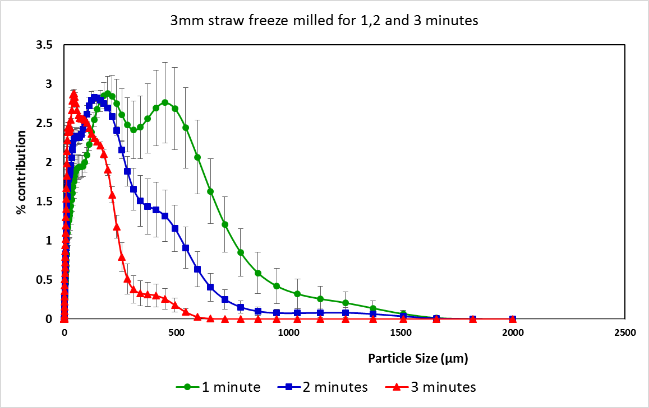
**
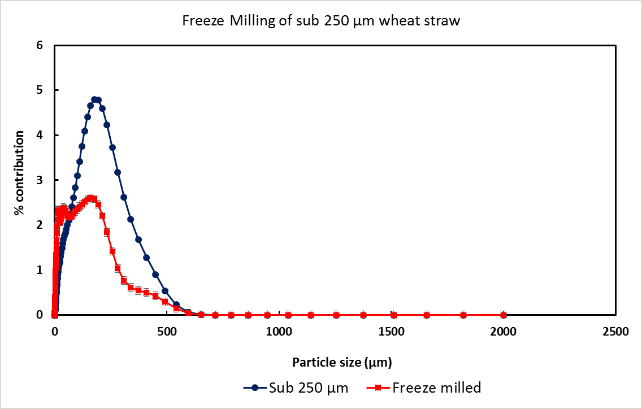
Figure S1: Particle size distributions of milled wheat straw**

(b)

(d)

(f)

(e)

(c)

(a)


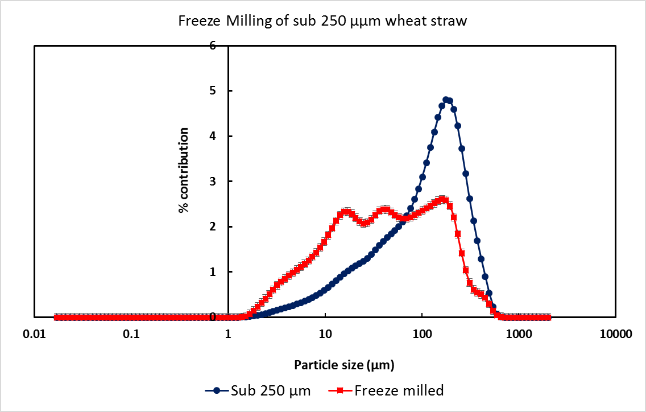

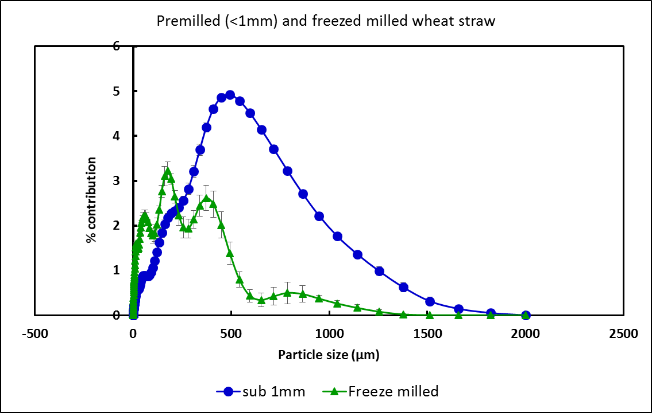

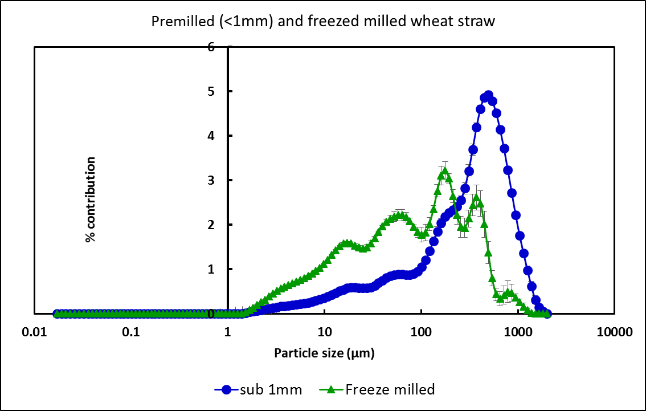

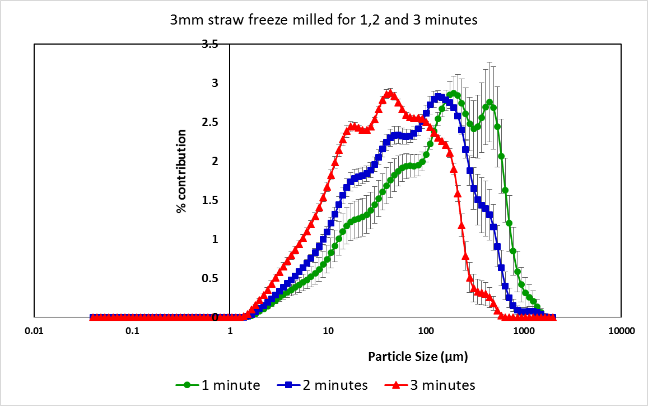

Supplement: Supplementary file 1 — Additional file 1: Figure S1. Particle size distributions of milled wheat straw. (a) [log scale for particle size] and (b) [linear scale for particle size]: 3 mm straw freeze milled for 1 (green circle), 2 (blue square) and 3 (red triangle) minutes at 8 cycles per second. (c) [log scale for particle size] and (d) [linear scale for particle size]: < 1 mm straw (blue circle) and after freeze milling for 1 min at 8 cycles per second (green triangle). (a) [log scale for particle size] and (b) [linear scale for particle size]: 3.6 g samples of < 0.25 mm straw before and after being pre-frozen and then subject to 5 × 3 min freeze milling cycles with an impact rate of 8 cycles per second. [file 13068_2017_914_MOESM1_ESM.docx]

**Supplementary Figure S2: Production of fermentation inhibitors (a) 2-FA and (b) 5-HMF**


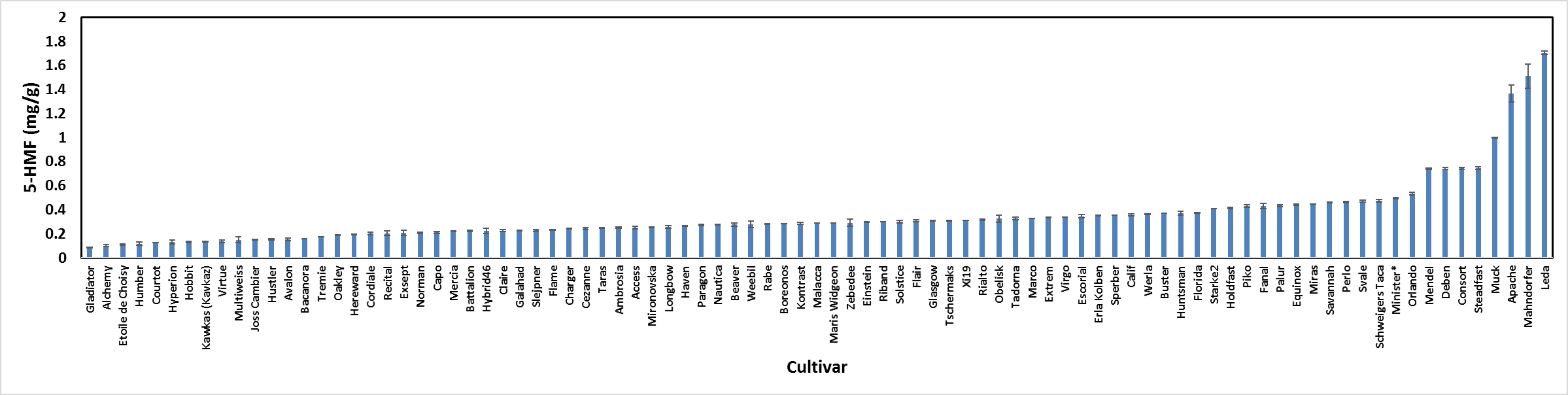


(a)


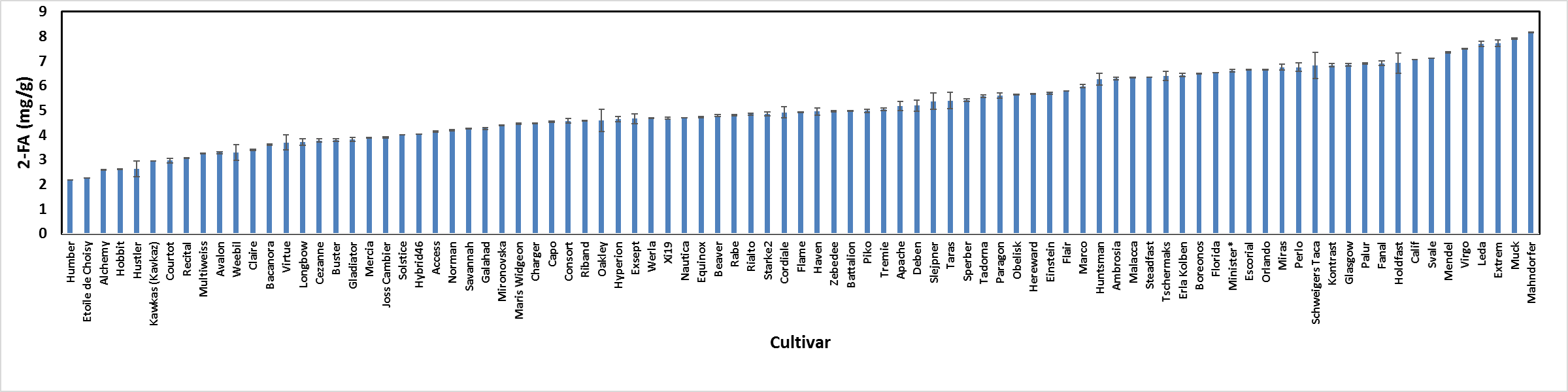


(b)

Supplement: Supplementary file 2 — Additional file 2: Figure S2. Production of fermentation inhibitors during pretreatment of 89 cultivars of wheat (a) 2-FA and (b) 5-HMF; means and standard deviations; n = 2. [file 13068_2017_914_MOESM2_ESM.docx]
