# Supplementary material for: Variation across a wheat genetic diversity panel for saccharification of hydrothermally pretreated straw
Source: Biotechnol Biofuels. 2017 Oct 2;10:227. doi: 10.1186/s13068-017-0914-x (PMC5625621; doi:10.1186/s13068-017-0914-x)

**Supplementary Figure S3: yields of saccharified glucose from straw of different wheat cultivars**


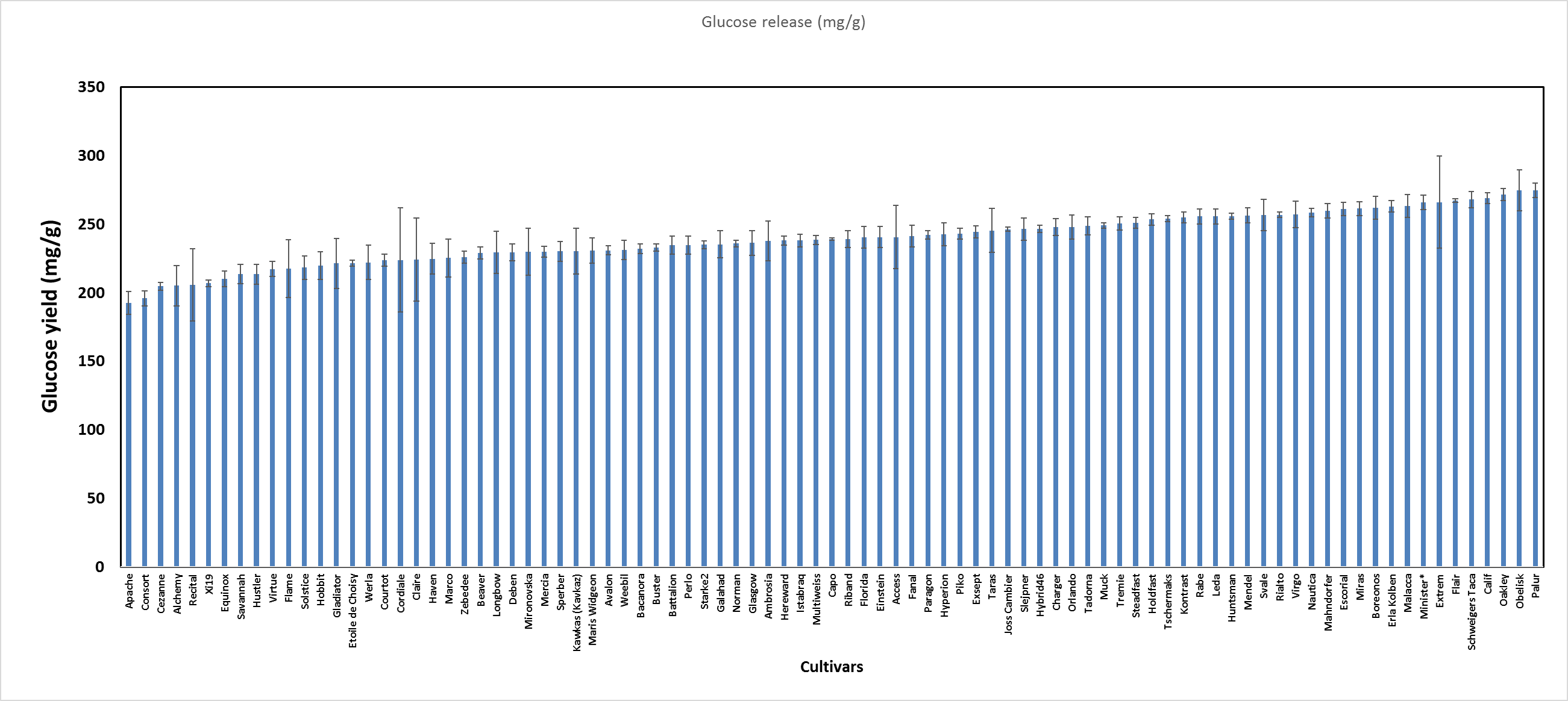

Supplement: Supplementary file 3 — Additional file 3: Figure S3. yields of saccharified glucose from pretreated straw of 89 different wheat cultivars; means and standard deviations; n = 2. [file 13068_2017_914_MOESM3_ESM.docx]
